# Editorial: The Cognitive Neuroscience of Visual Working Memory

**DOI:** 10.3389/fnsys.2017.00001

**Published:** 2017-01-19

**Authors:** Zsuzsa Kaldy, Natasha Sigala

**Affiliations:** ^1^Department of Psychology, University of Massachusetts BostonBoston, MA, USA; ^2^Brighton and Sussex Medical School, University of SussexBrighton, UK

**Keywords:** visual working memory, neuroimaging, development, prefrontal cortex, delay activity, fronto-parietal network, capacity, infants

Visual working memory (VWM) allows us to temporarily maintain and manipulate visual information in order to solve a task. The study of the brain mechanisms underlying this function began more than a half century ago, with Scoville and Milner's (1957) seminal discoveries with amnesic patients. As of 2016, more than 4000 studies have examined the brain mechanisms underlying VWM. In this Research Topic, our goal was to bring together perspectives on the cognitive neuroscience of VWM from multiple fields that have traditionally been fairly disjointed: human neuroimaging, electrophysiological and animal lesion studies, both in adults and in development.

The classic model of VWM posits that persistent delay activity in the prefrontal cortex is both sufficient and necessary to mediate visual working memory. Riley and Constantinidis contribute a thorough review of relevant primate studies, and provide compelling fresh evidence for it. They also survey a number of alternative models of VWM and conclude that each one can only mediate a limited range of memory-dependent behaviors. They also provide a detailed account of the tissue characteristics that make the prefrontal cortex (PFC) uniquely specialized to support this function.

Further support for the classic model is provided by Boschin and Buckley, who enhance it by offering an account of the functions of the frontopolar cortex (FPC) from a series of pioneering lesion and behavioral studies in the non-human primate. Specifically, they suggest that the FPC supports the exploration and evaluation of relative values of novel alternatives, some of which may turn out to be distractors, while the dorsolateral PFC maintains, manipulates, and selects relevant information, rules and strategies for the task at hand. Mansouri et al. review the role of VWM in executive control functions with an emphasis on abstract features, and representations of errors and conflicts in order to make adaptive behavioral adjustments. They note that primate performance in a Wisconsin Card Sorting Task analog is disrupted after lesions of the dorsolateral PFC, orbitofrontal cortex, but also of anterior cingulate cortex. Tsutsui et al. offer an integration of findings on visuospatial WM from two animal models: primates and rodents. Both lesion and single unit studies, together with anatomical patterns of fronto-parietal connectivity indicate that the dorsolateral PFC in the macaque is analogous in function to the medial PFC in the rat.

The alternative model of the PFC delay activity, which posits that it serves as a top-down signal that modulates posterior sensory areas, rather than it encodes stimulus information *per se* (see D'Esposito and Postle, 2015, for a recent review), has also received experimental support and is represented in this Research Topic. Desrochers et al. present data on the human and non-human primate rostrolateral PFC during error and conflict monitoring in task sequences. Lorenc et al.'s human neuroimaging study combines PFC disruption via TMS with behavioral data and multivariate analysis of fMRI data, and provides evidence for the causal role of PFC in top-down tuning of posterior sensory areas. This tuning was also shown to be dynamically changing according to current task goals.

Lara and Wallis critically review studies that report delay activity in the PFC as the neural correlate of VWM. They cast doubt on the claim that stimulus-relevant information is encoded in the PFC, and suggest long-range synchronization of oscillations as a candidate mechanism by which the PFC exerts top-down control on sensory neurons. Lee and Baker provide further evidence for the alternative model by reviewing imaging evidence for the topography of maintained information during VWM tasks. They conclude that VWM is a highly distributed process, and claim that the relevant information can be maintained in any of the systems involved in the initial stages of perceptual processing.

Wolff et al. contribute a proof-of-principle experimental EEG study that explores the possibility of exposing hidden states of VWM, employing a functional perturbation approach combined with multivariate decoding. Finally, Ambrose et al. tested the inter-individual stability of behavioral and neural VWM capacity measures. They found that while results from their two different tasks (an easier color vs. a harder shape VWM task) correlated within individuals both in behavior and in brain activity (BOLD response in the occipital and parietal cortices), there were no significant brain-behavior correlations in capacity. Both of these empirical findings open up a lot of questions for future work.

Let us now turn to the works in this Research Topic that examined VWM from a developmental perspective. In 2004, we presented a summary of what was then known about the early development of VWM in humans (Káldy and Sigala, 2004) and we also put forward a novel hypothesis. Building on the more recent alternative model of VWM organization in the adult brain that distinguishes between a fronto-parietal control network and more posterior information storage areas in the ventral visual stream, we hypothesized that young infants may rely more on the posterior areas when solving tasks that involve VWM. In the more than 10 years since the publication of that review, some significant progress has been made on the developmental emergence of VWM systems in the brain, but there are still a lot of open questions.

Fitch et al. surveys what is currently known about the emergence of these systems in the first five years of life. This mini-review concludes that both networks seem to be active before the end of the first year of life in humans, and a few pioneering studies have already identified VWM capacity-dependent neural activity in the occipital and parietal cortices of infants and young children.

Two empirical studies in this Research Topic that tested human infants found VWM-related activity in the ventral visual stream. Prior EEG studies have demonstrated that gamma-band power in the temporal cortex increases during periods while infants are maintaining an object representation in VWM (Kaufman et al., 2003, 2005). Here, Leung et al. have shown that this EEG signal increases with memory load (two objects vs. one). Optical imaging (fNIRS) studies reported by Wilcox and Biondi provided converging evidence. The occipital cortex (and posterior temporal cortex in younger infants) was involved during all events when infants had to maintain object representations in VWM. In addition to this, the anterior temporal cortex was selectively activated when infants maintained two distinct objects in VWM.

The medial temporal lobe (which includes the hippocampus, entorhinal, perirhinal, and parahippocampal cortices) has extensive connections with both frontal and temporal areas. Weiss et al. demonstrated the role of the perirhinal cortex in VWM development. They tested adult macaques that received neurotoxic lesions in the perirhinal cortex when they were 1–2 weeks old and found that these animals were impaired in VWM tasks that required trial-to-trial updating of visual information.

Two articles in our Research Topic have examined the complex interactions between visual attention and memory during development. Reynolds and Romano reviews the existing literature in infants, and conclude that while the role of sustained attention in long-term memory encoding has been well understood (see e.g., the now-classic works of Richards, 1997), the same is not true for relations between sustained attention and VWM performance in early development. They echo the conclusions of Fitch et al. that “future research should aim to examine relations between attention and working memory in infancy and early childhood using both psychophysiological and neural measures.”

We know more about attention-VWM interactions in older children, thanks to, among others, the EEG/ERP studies of Scerif and her colleagues. Shimi et al. investigated the magnitude of the N2pc in 10-year-old children and adults, and found that this neural signature of visual attention during the encoding phase of the task was related to their behavioral performance during the later recognition phase. This brain-behavior relationship was demonstrated on the individual level as well: children with large attentional cue benefits and high VWM capacity elicited an adult-like ERP response following attentional selection of the to-be-encoded item, whereas children with low VWM capacity did not.

In summary, this Research Topic includes nine up-to-date literature reviews and seven novel empirical studies approaching the neural mechanisms underlying visual working memory from human developmental, neuroimaging, and non-human mammalian perspectives. Together, they describe a common brain network that involves the fronto-parietal control system, various processing stages of the ventral visual stream, and the medial temporal lobe—with some differences in the weights and functions of the different structures. This extensive network seems to function in early infancy, and new multi-level approaches will help elucidate the details of the developmental trajectories.

## Author contributions

The two authors contributed equally to the preparation of this manuscript.

### Conflict of interest statement

The authors declare that the research was conducted in the absence of any commercial or financial relationships that could be construed as a potential conflict of interest.
